# The Immediate Treatment of Puerperal Sepsis.—I

**Published:** 1909-01-02

**Authors:** 


### Obstetrics.

### THE IMMEDIATE TREATMENT OF PUERPERAL SEPSIS.—I.

The successful treatment of puerperal infections very often, if not always, depends upon immediate recognition of the condition and prompt action. As the first symptom is usually a rise of temperature, and as infection is not the only cause of rises of temperature after labour, there is a tendency to put off investigation of the uterus and genital organs generally in the hope that the temperature may be the result of some accidental circumstance. It is true that constipation, mental emotions, breast disturbances, and intercurrent diseases, such as influenza, may cause rises of temperature during the puerperium. It is, however, a very safe maxim that infection of the genital organs should be first excluded by careful investigation before looking for some other cause for a rise of temperature. If this is made an absolute rule, puerperal infections will always be discovered at once, and much valuable time will be gained. The loss of a few hours may make a very serious difference to the patient, and even contribute to a fatal issue. It is clear, then, that a definite plan of campaign should be adopted at once in any case in which a rise of temperature indicates a possible genital infection. Even in modern times it is by no means definitely laid down in text-books of midwifery how such a case should be approached. The usual treatment adopted is a vaginal or an intra-uterine douche of a strong antiseptic without any preliminary thorough examination. Twenty-four hours is then allowed to elapse, and if the patient is no better the treatment is repeated. Drugs, such as quinine, are usually given at the same time, and the patient not uncommonly gets steadily worse. Nothing could be more futile than this haphazard method, and under such a *régime* it is not surprising that fatal cases occur.

In approaching such a case the following routine method is suggested. At the first serious rise of temperature a minute examination of the patient must be made, beginning with the genital system, and only when infection can be shown to be absent should other causes be looked for. On abdominal examination the height of the fundus uteri above the pubes must be noted after emptying the bladder, so as to judge whether involution is proceeding normally. In any uterine infection involution usually comes to a standstill, and the fundus will be too high. Tenderness of the uterus must be looked for, and is especially important in cases of decomposition of the uterine contents. The tenderness in such a case may be localised to one part of the uterus, and may perhaps be accounted for by adhesion of some contained material to one side or the other. Tenderness, however, may result from infection at the placental site without any decomposing contents. General abdominal tenderness apart from the uterus does not necessarily mean peritonitis, especially at this early stage; it is more likely to be the result of intestinal stasis and distension.

Next the vulva, perinæum, etc., must be inspected for lacerations—by no means uncommon sources of puerperal sepsis. If present these may present greyish or yellowish coloured exudates, or even sloughs, with much inflammatory redness and much pain on manipulation. If such wounds are found, and if the previous examination of the uterus has been negative, it is wise not to explore the uterus, but to treat the infected lacerations and wait 24 hours. The reason for this is obvious. If the uterus is not infected already, any attempt to explore it will be fraught with the greatest danger of carrying bacteria from the lacerated infected wound. Although lacerated wounds and uterus may be infected simultaneously, this is not common, and it is wiser not to risk carrying the infection upwards.

If, however, no infected wounds are found at the vaginal outlet, the next step is to note the character of the lochia, and finally to explore the interior of the uterus. As a rule in cases of decomposition of the uterine contents the lochia are offensive in the vagina; this, however, is not invariable. Cases occur sometimes in which the lochia on the pad or in the vagina are not offensive, and yet the interior of the uterus is full of stinking material. In such cases deodorisation occurs, and its explanation is not fully known. The amount of the lochia in most uterine infections is *increased* rather than diminished because of the subinvolution which accompanies the disease. The time-honoured legend that the lochia are suppressed in "puerperal fever," along with simultaneous suppression of mammary secretion, only holds good in the very rare cases of what may be called acutest septicæmia, in which no local lesions are found, and the patient may die as early as the third or fourth day. The lochia may also be altered in colour to brown, or almost black in cases where decomposition is occurring in the uterus.

To explore the uterus is the next step, and this investigation must be done in such a manner as not to convey fresh infection from outside, and also without hurting the patient. An anæsthetic may be required when there is any uterine tenderness, or more particularly when the perinæum is lacerated but not necessarily infected. The dorsal position is usually the most convenient for this purpose, and the hand must be most carefully prepared by scrubbing and disinfection before introduction. Indeed, it is better to wear a boiled thin rubber glove as the best means of protecting both parties. Before introducing the hand the vagina must be thoroughly washed out, or better, if under an anæsthetic, scrubbed out with cotton-wool held in forceps and soap and water, followed by an antiseptic. Then two fingers are introduced into the vagina (or the half hand if necessary), and one or two fingers are put into the uterus. The external hand presses down the fundus so that the internal fingers may explore every portion of the uterine cavity. By this means lacerations of the cervix or vagina may be detected, or retained decomposing materials found in the uterus. Sometimes all that can be found is a roughened and knobbly condition of the placental site.

